# Case Report: An Infant With Kabuki Syndrome, Alobar Holoprosencephaly and Truncus Arteriosus: A Case for Whole Exome Sequencing in Neonates With Congenital Anomalies

**DOI:** 10.3389/fgene.2021.766316

**Published:** 2021-11-25

**Authors:** Rishika P. Sakaria, Parul G. Zaveri, Shannon Holtrop, Jie Zhang, Chester W. Brown, Eniko K. Pivnick

**Affiliations:** ^1^ Department of Pediatrics, University of Tennessee Health Science Center, Memphis, TN, United States; ^2^ Le Bonheur Children’s Hospital, Memphis, TN, United States; ^3^ Department of Pathology and Laboratory Medicine, University of Tennessee Health Science Center, Memphis, TN, United States; ^4^ Department of Ophthalmology, University of Tennessee Health Science Center, Memphis, TN, United States

**Keywords:** KMT2D, kabuki syndrome (KS), holoprosencephaly (HPE), congenital anomalies, truncus, PAPVR, whole exome sequencing

## Abstract

Kabuki syndrome is a rare multiple anomalies syndrome associated with mutations in *KMT2D* or *KDM6A*. It is characterized by infantile hypotonia, developmental delay and/or intellectual disability, long palpebral fissures with everted lateral third of the lower eyelids and typical facial features. Intracranial anomalies occur infrequently in patients with KS and holoprosencephaly has only been recently described. Additionally, though congenital heart diseases are common in patients with KS, to our knowledge truncus arteriosus has never been reported in a patient with KS. We present an unusual case of KS in an infant with holoprosencephaly and truncus arteriosus with partial anomalous pulmonary venous return. Duo whole exome sequencing in our patient identified a pathogenic nonsense variant in exon 10 of *KMT2D* (c.2782C > T; p. Gln928*) establishing the diagnosis. This report further expands the phenotypic spectrum of patients with Kabuki syndrome and emphasizes the utility of performing large scale sequencing in neonates with multiple congenital anomalies.

## Introduction

Kabuki syndrome (KS) [OMIM# 147920 (KS1) and 300867 (KS2)] is a rare pleiotropic, monogenic syndrome that was first identified in 1981 in 10 Japanese patients (Kuroki et al., 1981; Niikawa et al., 1981). Variants in two different genes, lysine methyltransferase 2D (*KMT2D*) (Chr12q13.12) (55–80%) and lysine demethylase 6A (*KDM6A*) (Chr Xp11.2) (9–14%) were later identified to be causative for KS (Ng et al., 2010; Lederer et al., 2012; Miyake et al., 2013; Dentici et al., 2015). KS is a clinically recognizable syndrome consisting of postnatal growth retardation, distinctive facial features (long palpebral fissures with eversion of the lateral third of the lower eyelids, arched and broad eyebrows with sparseness or notching of the lateral third of the brows, large prominent ears, and short *columella* with depressed nasal tip), skeletal anomalies, dermatoglyphic abnormalities, persistent fingertip pads and intellectual disability (ID); however, many of the characteristic facial features are not clearly evident in early infancy (Adam et al., 2019).

Several structural brain anomalies have been recently reported in patients with KS. In 2019, 3 cases of KS1 with holoprosencephaly (HPE) were reported for the first time. Although congenital heart defects (CHDs) have been reported in 70% of patients with KS1, only one of the three cases of KS1 with HPE had right ventricular dilatation and hypertrophy and none of them were reported to have structural heart defects (Tekendo-Ngongang et al., 2019; Daly et al., 2020). Additionally, no patient with KS has been reported to have truncus arteriosus (TA). We describe an unusual association of TA and HPE in a patient with KS1 and review the intracranial structural anomalies associated with KS to date.

## Case Description

The patient was a female infant born at 32 weeks of gestation. The mother was 19 and the father was 25 at the time of birth. The mother previously had one healthy daughter with a different partner. Prenatal history was significant for late and limited prenatal care in second trimester and lack of folic acid supplementation during the first trimester. The mother denied history of drugs, tobacco or alcohol use. There was no history of diabetes mellitus. Per mother, the facial features of the fetus were not well-visualized during any of the prenatal ultrasounds. The patient was born via precipitous vaginal delivery. Her birthweight was 1.68 kg (<1st percentile) with a head circumference of 27.7 cm (22nd percentile) and length 41 cm (46th percentile). She was initially placed on non-invasive ventilation. Her oxygen saturation remained below 90% despite 100% oxygen. This prompted an echocardiogram which revealed a large ventricular septal defect (VSD), a small atrial septal defect (ASD), probable mitral atresia and probable D-transposition of great arteries (TGA) with adequate ventricular function. Chest X-ray at the outside hospital showed a cystic lesion in the right upper lobe which was concerning for a cystic-adenomatoid malformation (CCAM) pushing the heart inferiorly. She was intubated and transferred to our neonatal intensive care unit (NICU) on day of life two for higher level of care.

**FIGURE 1:**
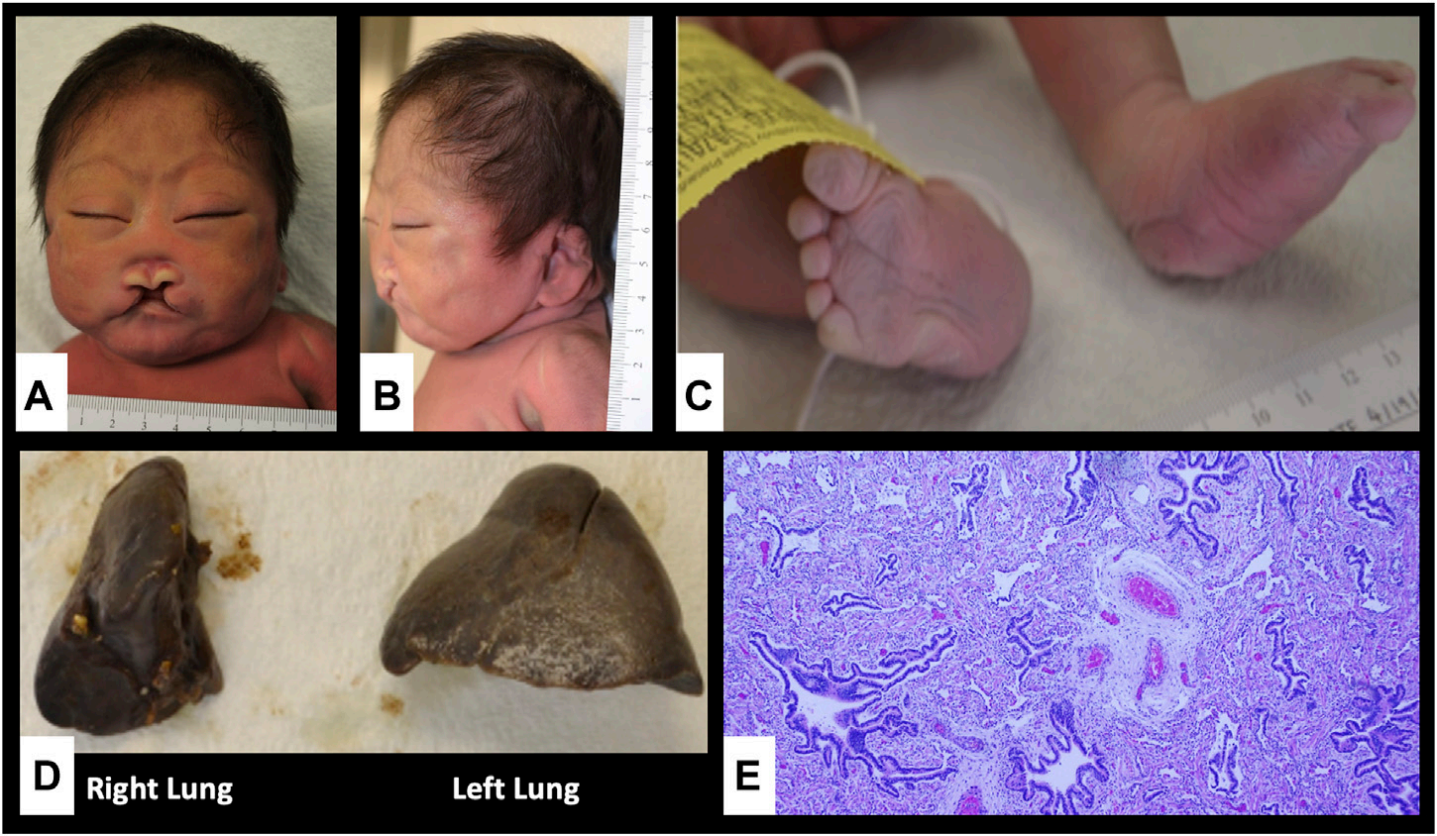
Autopsy findings showing craniofacial dysmorphism, limb abnormalities and lung hypoplasia. **(A,B)** Flat face, low anterior and posterior hairlines, synophrys, hypotelorism, upslanting palpebral fissures, large floppy low-set ears, large midline cleft lip and palate, absent nasal septum, large mouth with down-turned corners and micrognathia; **(C)** Rocker bottom feet and right talipes equinovalgus; **(D)** Unilobular right lung; **(E)** pulmonary hypoplasia and congenital pulmonary airway malformation, type II in the right lung (Hematoxylin and Eosin stain, x40).

**FIGURE 2:**
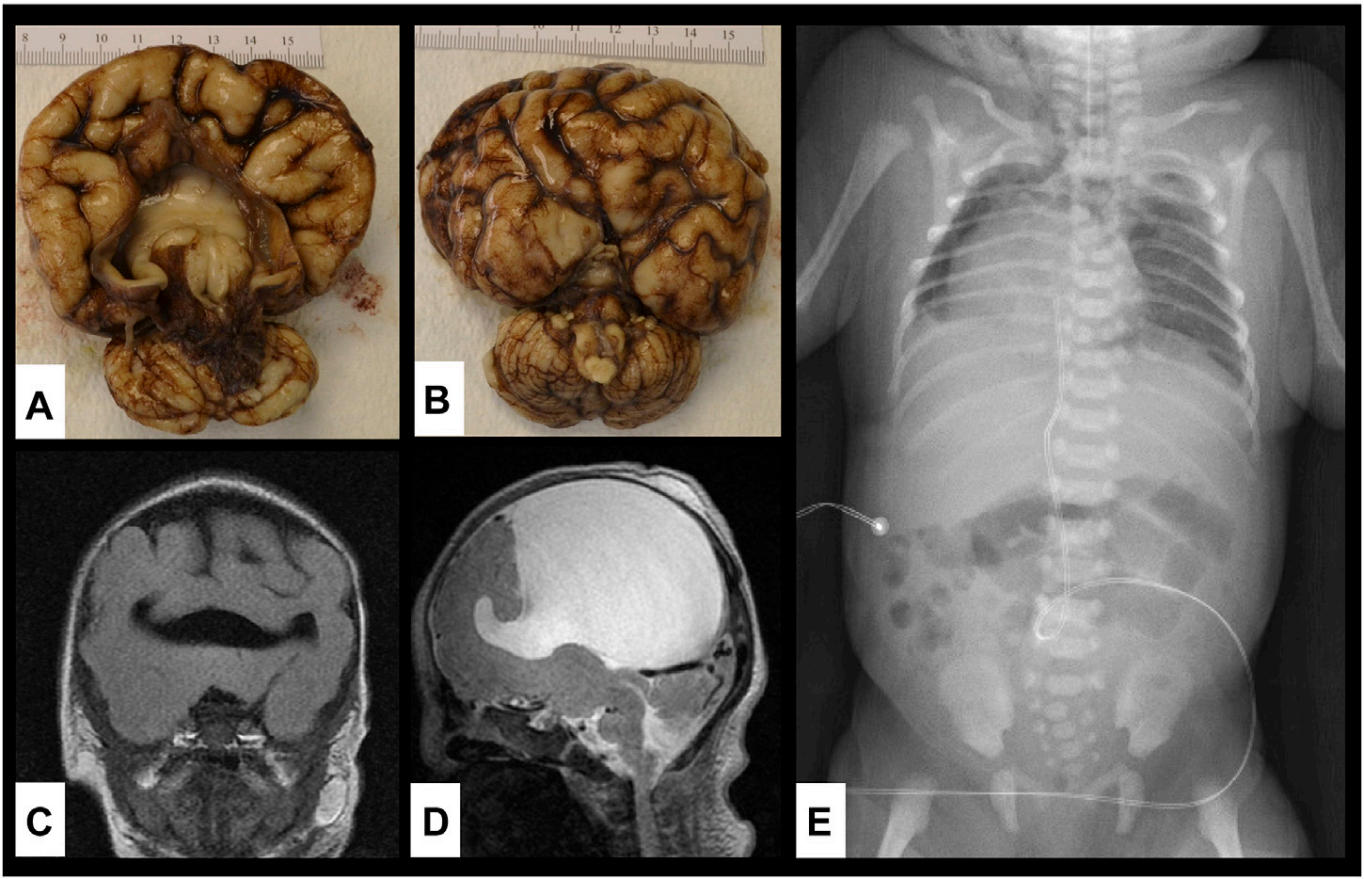
Brain and vertebral abnormalities. **(A,B)** Gross specimen of the brain on autopsy showing alobar holoprosencephaly with disordered gyral pattern, a single common ventricle, a large dorsal cyst (contained 85 ml cerebrospinal fluid) and absence of the olfactory tracts and bulbs; **(C,D)** MRI of the brain showing single ventricle and alobar holoprosencephaly; **(E)** X-ray of chest and abdomen showing dextroposition of the heart, segmentation abnormalities of multiple thoracic vertebrae and absent left lateral portion of S2 vertebra.

On admission, her examination was significant for midline cleft lip and palate, hypotelorism, up-slanting palpebral fissures, absent nasal septum, large, prominent ears, clinodactyly, a single flexion crease of the right fifth digit, a single transverse palmar crease on the left, rocker-bottom feet with talipes equinovalgus, anterior anus, sacral dimple, possible craniosynostosis and a single umbilical artery (Figure 1). X-ray on admission showed segmentation abnormalities of the thoracic spine with absence of the left lateral portion of S2 (Figure 2). CT-angiography of the chest revealed type A2 truncus arteriosus with hypoplastic left ventricle and VSD, partial anomalous pulmonary venous return (PAPVR) and septate cystic structure replacing the right upper lobe extending across the midline to the contralateral side, consistent with CCAM. Ultrasound of the abdomen was significant for an ectopic right, hypoplastic, pelvic kidney. MRI of the brain was consistent with alobar HPE and craniosynostosis (Figure 2).

Due to multiple, complex congenital anomalies, the parents decided to withdraw life-sustaining measures. The infant died at 8 days of age.

In addition to the above findings, autopsy further showed absence of olfactory tracts and bulbs, diffuse cortical dysplasia, absence of the nasal bone, small unilobular right lung with type II congenital pulmonary airway malformation (CPAM) and abnormal right diaphragm with partial liver herniation (Figure 1).

## Molecular Analysis

Blood was sent for chromosomal microarray (CMA) (Invitae, Irvine, CA, United States) which revealed a normal female genomic profile with multiple regions of homozygosity (ROHs) (Supplementary Table S1). We searched every ROH to determine if there were strong candidate autosomal recessive disease genes that fit the patient’s clinical features (https://genescout.omim.org). The ROH on chromosome 2 contained *KYNU*, which is associated with vertebral, cardiac, renal and limb defects syndrome 2 (VCRL2; https://omim.org/entry/617661), which includes some of the features seen in our patient but not all. We also investigated the homozygous regions for syndromes of uniparental disomy (UPD). Of the chromosomes with ROH, imprinting disorders have been associated with UPD of chromosomes 6, 11, 15 and 16 (Eggermann et al., 2015). Maternal UPD [upd(6)mat; upd(16)mat] and paternal UPD [upd(6)pat] have all been associated with IUGR and upd(6)pat also causes transient neonatal diabetes, macroglossia and omphalocele (Eggermann et al., 2015). While the patient did have IUGR, her phenotype cannot be fully explained by upd(6)pat, upd(6)mat, or upd(16)mat. Similarly, the patient’s facial features, holoprosencephaly and conotruncal anomaly are not consistent with Beckwith-Wiedemann syndrome [upd(11p15)pat], Prader-Willi syndrome [upd(15)mat] or Angelman syndrome [upd(15)pat].

Duo whole exome sequencing (WES) (Perkin Elmer Genomics, Pittsburg, PA, United States) was performed on patient’s and mother’s blood. We were unable to obtain a paternal sample. The following phenotypic terms were applied for analysis: ASD, VSD, dextro-transposition of the great arteries, hypoplastic left heart, PAPVR, pulmonary arterial hypoplasia, possible pneumomediastinum, pneumothorax, cystic abnormality of upper lobe of right lung, possible congenital cystic adenomatoid malformation or congenital pulmonary airway malformation, alobar holoprosencephaly, microcephaly, craniosynostosis, ectopic pelvic right kidney, sacral dimple, thoracic vertebral abnormalities, malformation of S2 vertebra, anteriorly displaced anus, hypotelorism, upslanting palpebral fissures, vascular flat marking on forehead, cleft lip and palate, hypoplastic nasal bone and low-set ears. 99.37% of target bases in disease causative genes were fully covered. The average coverage per target base was 135.54. A complete list of genes analyzed is provided in Supplementary Table S2. A heterozygous, stop-gain variant (not maternally inherited) in *KMT2D* (NM_003482.3) c.2782C > T (p.Gln928Ter) was identified in exon 10, which leads to replacement of the glutamine at amino acid position 928 with a termination codon. This variant was classified as pathogenic for KS1 based on the American College of Medical Genetics (ACMG) guidelines. This classification was supported using the following criteria: PVS1 (the variant is predicted to cause nonsense mediated decay in a biologically relevant transcript), PM2 (the variant is not found in general population), a half point for PM1 (the premature stop codon caused by the variant causes approximately 83% of the protein to be lost, which includes all five binding sites and domains, and *KMT2D* is a single transcript gene) and another half point for PS2 (the variant was not maternally inherited, but the father was unable to be tested; thus, the variant is presumed (not confirmed) to be *de novo*). This *KMT2D* variant has been reported once in literature in a patient with Kabuki syndrome who did not have HPE (Murakami et al., 2020). No pathogenic variants or variants of unknown significance in *KYNU* were identified by WES.

## Discussion

KS is a rare, multisystem congenital anomalies syndrome with an estimated prevalence of 1 in 32,000. It is caused due to mutations affecting *KMT2D* (autosomal dominant) or *KDM6A* (X-linked dominant) and to date over 500 cases of genetically confirmed KS have been reported in literature. The 2019 international consensus criteria for definitive diagnosis of KS, require a history of infantile hypotonia, developmental delay and/or ID with pathogenic/likely pathogenic variant in *KMT2D* or *KDM6A* and/or typical dysmorphic features (long palpebral fissures with eversion of the lateral third of the lower eyelid and at least two of the following features: arched and broad eyebrows with the lateral third displaying notching or sparseness; short *columella* with depressed nasal tip; large prominent/cupped ears; and persistent fingertip pads) (Adam et al., 2019). However, the diagnosis of KS is often difficult to establish in neonates, where the typical features of KS may not yet be recognizable (Vaux et al., 2005). Our patient had long, up-slanting palpebral fissures but eversion of the lower eyelids was not apparent.

*KMT2D* and *KDM6A* are interacting members of the histone methyl transferase family that participate in chromatin remodeling, thereby impacting the transcription of a variety of downstream genes (Demers et al., 2007; Sobreira et al., 2017). Reduced expression of orthologs *kmt2d*, *kdm6a* and *kdm6al* in Zebrafish has been shown to cause brain, cardiac and craniofacial abnormalities, correlating with the phenotype of KS (Van Laarhoven et al., 2015).

The incidence of structural brain defects in KS is unknown. We performed a systematic PubMed search with key words “Kabuki,” “KMT2D” and “KDM6A” each with “brain,” “craniofacial” and “central nervous.” Only articles in English language were used. We found 40 papers describing 68 patients with KS and structural brain anomalies. Of the 66 patients, 31 were reported after 2012 and had a molecular diagnosis of KS. Thus, the incidence of brain anomalies reported in patients with KS is predicted to be <10%. The spectrum of structural intracranial malformations and associated cardiac malformations reported in KS is provided in Table 1 and Supplementary Table S3. Most frequently reported brain malformations include ventriculomegaly/hydrocephalus and delayed myelination. *KMT2D* variants have been previously reported in five patients with HPE. In two of the five patients, there was no description of the patients’ other clinical features and it was unknown if the *KMT2D* variants were pathogenic, one patient had semi-lobar HPE and two had alobar HPE (Solomon et al., 2018; Daly et al., 2020; Tekendo-Ngongang et al., 2019).

**TABLE 1:** Intracranial malformations in patients with molecularly confirmed Kabuki Syndrome and associated congenital heart defects.

*Malformations*	*Cases with genetic diagnosis of KS* (*Total = 32* *^a^*) *N* *^b^*(*%*)
*Genes Affected*	
KMT2D	13^c^ (41)
KDM6A	19 (59)
*Intracranial Malformations*	
*Hydrocephalus/Ventriculomegaly*	8 (25)
*Agenesis/Dysgenesis of corpus callosum*	4 (13)
*Holoprosencephaly*	4^c^ (13)
*Arnold-Chiari Malformations*	1 (3)
*Dandy-Walker malformation/Cerebellar Vermis hypoplasia*	3 (9)
*Adenohypophysis hypoplasia*	3 (9)
*Cerebral atrophy/polymicrogyria*	2 (6)
*Delayed myelination*	5 (16)
*Pituitary microadenoma*	1 (3)
*Periventricular leukomalacia*	2 (6)
*Absent olfactory bulb and tracts*	1^c^ (3)
*Congenital Heart Defects*	**17** ^c^ **(53)**
*Coarctation of Aorta*	5 (16)
*Aortic stenosis*	2 (6)
*Bicuspid Aortic valve*	2 (6)
*Atrial septal defect*	4^c^ (13)
*Ventricular septal defect*	3^c^ (9)
*Partially anomalous pulmonary venous return*	1^c^ (3)
*Truncus arteriosus*	1^c^ (3)

The bold values represent the total number of congenital heart defects. The values provided below are types of congenital heart defects.

a Including present case.

b Not mutually exclusive.

c Present in our patient.

About a third of the patients with HPE have chromosomal anomalies. Trisomy 13 is the most common followed by trisomy 18 and triploidy. Mutations in other genes have also been associated with HPE, most commonly *SHH, ZIC2, SIX3* and *TGIF* (Ramakrishnan and Gupta, 2021)*.* CMA is often sent as first-line genetic testing in patients with HPE followed by commercially available HPE panels. However, CMA cannot detect single nucleotide changes and HPE panels do not test for mutations in *KMTD2* or *KDM6A*. In this patient, in the absence of typical features of KS, the diagnosis was possible only due to the detection of the *KMT2D* mutation by WES. We suspect that HPE spectrum disorders may be more common in KS than previously realized.

Congenital heart defects (CHD) have been reported to occur in up to 80% of patients with KS-septal defects are the most common followed by coarctation of the aorta and bicuspid aortic valve (Digilio et al., 2001; Digilio et al., 2017). Conotruncal defects have been reported to occur in 8% cases of molecularly confirmed KS (Double outlet right ventricle (2%) tetralogy of Fallot (2%), infundibular stenosis (2%) and interrupted aortic arch (*n* = 1). TA has never been reported to occur in a patient with Kabuki syndrome. Of the 32 patients with molecularly confirmed KS who had structural brain anomalies (including the present case), 53% were found to have CHD (Table 1).

To our knowledge, this is the first case of *KMT2D* mutation associated with TA. The *KMT2D* histone methyltransferase has been shown to control neural crest cell (NCC) differentiation and facial morphology (Shpargel et al., 2020). NCCs and disruption of neural tube development have been associated with TA (Keyte and Hutson, 2012).

Although we cannot absolutely determine whether HPE and TA are part of the KS phenotypic spectrum or if they occur coincidentally, we did not identify any other genetic/environmental cause of HPE or TA in this patient, and patients with Kabuki syndrome have presented with features that fall within either the HPE or conotruncal spectrum. Unlike neural tube defects, folic acid supplementation has not been shown to protect against HPE (Petryk et al., 2015). It is possible that with increasing use of WES or whole genome sequencing (WGS), more patients with *KMT2D*/*KDM6A* pathogenic mutations with HPE and/or TA and other conotruncal abnormalities will be identified. This case further illustrates the clinical utility of WES, providing an answer that would not have been attainable given the absence of important, defining clinical features early in life. Establishing the diagnosis helped to provide closure for the family and informed recurrence risk for future pregnancies. In general, WES and WGS also provide important information regarding prognosis and surveillance measures and inform clinical management decisions (Meng et al., 2017; Clark et al., 2018; Manickam et al., 2021; NICUSeq study group et al., 2021).

## Conclusion

As many of the classical features of KS are not apparent until later in life, neonates with KS with critical congenital anomalies may remain undiagnosed clinically. KS should be included in the initial differential diagnosis of all neonates with HPE and/or conotruncal heart defects, as the association with KS might be higher than previously recognized. Early WES or WGS in the neonatal period may help identify more cases of KS, further expand the neonatal phenotype of this rare syndrome and help the medical team to appropriately counsel families.

## Data Availability

The original contributions presented in the study are included in the article/Supplementary Material, further inquiries can be directed to the corresponding author.

## Supplementary Material

Supplementary Table S1 Regions of homozygosity identified in the patient.

Supplementary Table S2 List of genes pulled by the phenotypic filter and analyzed in the whole exome sequencing.

Supplementary Table S3 Patients with Kabuki syndrome and structural brain abnormalities reported in literature.
