# Supplementary material for: Case Report: An Infant With Kabuki Syndrome, Alobar Holoprosencephaly and Truncus Arteriosus: A Case for Whole Exome Sequencing in Neonates With Congenital Anomalies
Source: Front Genet. 2021 Nov 25;12:766316. doi: 10.3389/fgene.2021.766316 (PMC8660850; doi:10.3389/fgene.2021.766316)
Supplement: Supplementary file 1 [file DataSheet2.PDF]

Supplementary table 1: Regions of homozygosity detected with chromosomal microarray

**Regions of homozygosity (GRCh17/hg19)**	**Base pairs (Mb)**
Chr1:26941213-29673089	2.73
Chr1:50592601-53083115	2.49
Chr2:143612415-145725877	2.11
Chr3:48754842-52236624	3.48
Chr5:118183823-120901367	2.72
Chr6:47161888-50165400	3.00
Chr8:46847101-49993085	3.15
Chr8:111187715-114461787	3.27
Chr10:110724030-113062001	2.34
Chr11:63164238-67274465	4.11
Chr17:14587022-22261706	7.67
Chr17:25294773-32700009	7.41
Chr15:82425034-84592782	2.17
Chr16:46392788-48933830	2.45
