# Supplementary material for: Case Report: An Infant With Kabuki Syndrome, Alobar Holoprosencephaly and Truncus Arteriosus: A Case for Whole Exome Sequencing in Neonates With Congenital Anomalies
Source: Front Genet. 2021 Nov 25;12:766316. doi: 10.3389/fgene.2021.766316 (PMC8660850; doi:10.3389/fgene.2021.766316)
Supplement: Supplementary file 2 [file DataSheet4.PDF]

Supplementary table 3: Patients with Kabuki syndrome and structural brain abnormalities.

Reference	Year	Cranial Malformation	Heart defect	Gene involved	Genetic change	Predicted protein change
**Ito et al**	2013	Absence of high intensity signal of posterior pituitary lobe	VSD	*KMT2D*	c.8813C>T,<br>(Paternally inherited, 0.55% frequency in general population)	p.Pro2938Leu
**Lindgren et al**	2013	Cerebral atrophy with mild ventriculomegaly	-	*KDM6A*	46,X,t(X;5)(p11.3;q35.3)<br>inv(5)(q35.3q35.1)dn	
**Cheon et al**	2014	Arnold Chiari malformation	ASD	*KDM6A*	c.3876_3878delTAA+1delG	
**Pietzner et al**	2014	Non-specific small spotty signal intensity increases at the anterior horn and a delay in myelination	-		46,X+mar1 chromosome	
**Banka et al**	2015	Delayed myelination	Abnormal Aorta	*KDM6A*	c.3878 + 3_3878 + 6delAAGT	
		Cerebellar vermis hypoplasia	-	*KDM6A*	c.3548+2T>C	
		Septum pellucidum and white matter hypoplasia	VSD, subaortic membrane	*KDM6A*	exon6 deletion	
**Bögershausen et al**	2016	multifocal hemorrhagic lesions superior to the frontal horn of the left lateral ventricle.	-	*KMT2D*	c.10588delC	p.(Glu3530Serfs*128)
**Guo et al**	2018	Congenital hydrocephalus	-	*KDM6A*	c.335-1G > T	
**Porntaveetus et al**	2018	Perientricular leukomalacia	-	*KDM6A*	c.327_333delTCCAAAA	p.Tyr109*
**Shangguan et al**	2019	Delayed myelination	VSD, PDA, PFO	*KDM6A*	c.2668-2671del	p.N891Vfs*27
		Thinning of the pituitary	-	*KMT2D*	c.16442delG	p.C5481Lfs*6)
**Tekendo-Ngongang et al**	2019	Alobar holoprosencephaly	-	*KMT2D*	c.12565G>T	p.Gly4189*
		Alobar holoprosencephaly	RV hypertrophy and dysfunction	*KMT2D*	c.5A>G	p.Asp2Gly
**Yap et al**	2019	Congenital partial agenesis of the corpus callosum with posterior interhemispheric cyst	small ASD, Biventricular hypertrophy	*KDM6A*	exon 3-24 deletion	
**Bruni et al**	2020	Thinning of the Corpus callosum	-	*KMT2D*	c.2413C> T	p. His805Tyr
**Daly et al**	2020	Lobar holoprosencephaly		*KMT2D*	c.6295C > T	p.R2099X

**Guo et al**	2020	4th ventricular enlargement and cerebellar vermis hypoplasia (Dandy Walker variant)	PDA, PFO, tricuspid regurgitation	*KMT2D*	c.12165del	p.(Glu4056Serfs*10)
**Di Candia et al**	2021	Upper parietal gyrus reduction, enlarged ventricles		*KDM6A*	c.2326G>T	p.Asp776Tyr
		Adenohypophysis hypoplasia, empty sella	-	*KDM6A*	c.975-1G>A (r.876_1320del)	p.Cys293Ilefs*26
		Ischemia outcomes	VSD, Coarctation of Aorta	*KMT2D*	c.5575delG	p.Asp1859Thrfs*17
		Enlarged lateral ventricles	VSD, PFO	*KMT2D*	c.6595delT	p.Tyr2199Ilefs*65
		Corpus callosum dysmorphism	VSD, Coarctation of Aorta	*KMT2D*	c.3161_3171delCGTTGAGTCCC	p.Pro1054Hisfs*10
		Pituitary microadenoma	Aortic valve dysplasia	*KMT2D*	c.7891C>T	p.Gly2631*
**Faundes et al**	2021	Delayed CNS myelination	ASD	*KDM6A*	c.514C>T	p.Arg172*
		Cerebral infarction		*KDM6A*	c.1846dupA	p.Thr616AsnfsTer5
		Ventriculomegaly	Coarctation of Aorta	*KDM6A*	c.1924-1G>C	
		Periventricular Leukomalacia, Dandy-Walker malformation, Intracranial hemorrhage	Coarctation of Aorta, Bicuspid aortic valve, Aortic stenosis, Mitral stenosis	*KDM6A*	c.2150dupA	p.Ser718GlufsTer12
		Ventriculomegaly	Coarctation of Aorta, PDA	*KDM6A*	c.3062G>A	p.Trp1021*
		Dysgenesis of corpus callosum, Ventriculomegaly, Defect of septum pellucidum		*KDM6A*	c.3238_3249delinsGGGTCC	p.Lys1080_Lys1083delinsGlySer
		Delayed CNS myelination, Solitary focal T2 hyperintensity in left frontal subcortical white matter	Bicuspid aortic valve, Aortic stenosis	*KDM6A*	c.3668G>A	p.Gly1223Asp
**This report**	2021	Alobar holoprosencephaly	Type A2 Truncus arteriosus with hypoplastic LV, PAPVR, VSD, ASD	*KMT2D*	c.2782C>T	p.Gln928*

VSD, ventricular septal defect; ASD: atrial septal defect; PDA, patent ductus arteriosus; PFO, patent foramen ovale; RV, right ventricle; CNS, central nervous system; LV, left ventricle; PAPVR, partially anomalous pulmonary venous return.

References for this table:

Banka, S., Lederer, D., Benoit, V., Jenkins, E., Howard, E., Bunstone, S., Kerr, B., McKee, S., Lloyd, I. C., Shears, D., Stewart, H., White, S. M., Savarirayan, R., Mancini, G. M., Beysen, D., Cohn, R. D., Grisart, B., Maystadt, I., & Donnai, D. (2015). Novel KDM6A (UTX) mutations and a clinical and molecular review of the X-linked Kabuki syndrome (KS2). *Clinical genetics*, *87*(3), 252–258. https://doi.org/10.1111/cge.12363

Bögershausen, N., Altunoglu, U., Beleggia, F., Yigit, G., Kayserili, H., Nürnberg, P., Li, Y., Altmüller, J., & Wollnik, B. (2016). An unusual presentation of Kabuki syndrome with orbital cysts, microphthalmia, and cholestasis with bile duct paucity. *American journal of medical genetics. Part A*, *170*(12), 3282–3288. https://doi.org/10.1002/ajmg.a.37931

Bruni, V., Scozzafava, C., Gnazzo, M., Parisi, F., Sestito, S., Pensabene, L., Novelli, A., & Concolino, D. (2021). Facial Dysmorphisms, Macrodontia, Focal Epilepsy, and Thinning of the Corpus Callosum: A Rare Mild Form of Kabuki Syndrome. *Journal of pediatric genetics*, *10*(1), 49–52. https://doi.org/10.1055/s-0040-1701645

Cheon, C. K., Sohn, Y. B., Ko, J. M., Lee, Y. J., Song, J. S., Moon, J. W., Yang, B. K., Ha, I. S., Bae, E. J., Jin, H. S., & Jeong, S. Y. (2014). Identification of KMT2D and KDM6A mutations by exome sequencing in Korean patients with Kabuki syndrome. *Journal of human genetics*, *59*(6), 321–325. https://doi.org/10.1038/jhg.2014.25

Daly, T., Roberts, A., Yang, E., Mochida, G. H., & Bodamer, O. (2020). Holoprosencephaly in Kabuki syndrome. *American journal of medical genetics. Part A*, *182*(3), 441–445. https://doi.org/10.1002/ajmg.a.61454

Di Candia, F., Fontana, P., Paglia, P., Falco, M., Rosano, C., Piscopo, C., Cappuccio, G., Siano, M. A., De Brasi, D., Mandato, C., De Maggio, I., Squeo, G. M., Monica, M. D., Scarano, G., Lonardo, F., Strisciuglio, P., Merla, G., & Melis, D. (2021). Clinical heterogeneity of Kabuki syndrome in a cohort of Italian patients and review of the literature. *European journal of pediatrics*, 10.1007/s00431-021-04108-w. Advance online publication. https://doi.org/10.1007/s00431-021-04108-w

Faundes, V., Goh, S., Akilapa, R., Bezuidenhout, H., Bjornsson, H. T., Bradley, L., Brady, A. F., Brischoux-Boucher, E., Brunner, H., Bulk, S., Canham, N., Cody, D., Dentici, M. L., Digilio, M. C., Elmslie, F., Fry, A. E., Gill, H., Hurst, J., Johnson, D., Julia, S., … Banka, S. (2021). Clinical delineation, sex differences, and genotype-phenotype correlation in pathogenic KDM6A variants causing X-linked Kabuki syndrome type 2. *Genetics in medicine : official journal of the American College of Medical Genetics*, *23*(7), 1202–1210. https://doi.org/10.1038/s41436-021-01119-8

Guo, Z., Liu, F., & Li, H. J. (2018). Novel KDM6A splice-site mutation in kabuki syndrome with congenital hydrocephalus: a case report. *BMC medical genetics*, *19*(1), 206. https://doi.org/10.1186/s12881-018-0724-4

Guo, W., Zhao, Y., Li, S., Wang, J., & Liu, X. (2020). Hypoglycemia and Dandy-Walker variant in a Kabuki syndrome patient: a case report. *BMC medical genetics*, *21*(1), 193. https://doi.org/10.1186/s12881-020-01117-8

Ito, N., Ihara, K., Tsutsumi, Y., Miyake, N., Matsumoto, N., & Hara, T. (2013). Hypothalamic pituitary complications in Kabuki syndrome. *Pituitary*, *16*(2), 133–138. https://doi.org/10.1007/s11102-012-0386-8

Lindgren, A. M., Hoyos, T., Talkowski, M. E., Hanscom, C., Blumenthal, I., Chiang, C., Ernst, C., Pereira, S., Ordulu, Z., Clericuzio, C., Drautz, J. M., Rosenfeld, J. A., Shaffer, L. G., Velsher, L., Pynn, T., Vermeesch, J., Harris, D. J., Gusella, J. F., Liao, E. C., & Morton, C. C. (2013). Haploinsufficiency of KDM6A is associated with severe psychomotor retardation, global growth restriction, seizures and cleft palate. *Human genetics*, *132*(5), 537–552. https://doi.org/10.1007/s00439-013-1263-x

Pietzner, V., Weigel, J. F., Wand, D., Merkenschlager, A., & Bernhard, M. K. (2014). Low-level hyperinsulinism with hypoglycemic spells in an infant with mosaic Turner syndrome and mild Kabuki-like phenotype: a case report and review of the literature. *Journal of pediatric endocrinology & metabolism : JPEM*, *27*(1-2), 165–170. https://doi.org/10.1515/jpem-2013-0090

Porntaveetus, T., Abid, M. F., Theerapanon, T., Srichomthong, C., Ohazama, A., Kawasaki, K., Kawasaki, M., Suphapeetiporn, K., Sharpe, P. T., & Shotelersuk, V. (2018). Expanding the Oro-Dental and Mutational Spectra of Kabuki Syndrome and Expression of *KMT2D* and *KDM6A* in Human Tooth Germs. *International journal of biological sciences*, *14*(4), 381–389. https://doi.org/10.7150/ijbs.23517

Shangguan, H., Su, C., Ouyang, Q., Cao, B., Wang, J., Gong, C., & Chen, R. (2019). Kabuki syndrome: novel pathogenic variants, new phenotypes and review of literature. *Orphanet journal of rare diseases*, *14*(1), 255. https://doi.org/10.1186/s13023-019-1219-x

Tekendo-Ngongang, C., Kruszka, P., Martinez, A. F., & Muenke, M. (2019). Novel heterozygous variants in KMT2D associated with holoprosencephaly. *Clinical genetics*, *96*(3), 266–270. https://doi.org/10.1111/cge.13598

Yap, K. L., Johnson, A., Fischer, D., Kandikatla, P., Deml, J., Nelakuditi, V., Halbach, S., Jeha, G. S., Burrage, L. C., Bodamer, O., Benavides, V. C., Lewis, A. M., Ellard, S., Shah, P., Cody, D., Diaz, A., Devarajan, A., Truong, L., Greeley, S., De Leó-Crutchlow, D. D., … Del Gaudio, D. (2019). Congenital hyperinsulinism as the presenting feature of Kabuki syndrome: clinical

and molecular characterization of 9 affected individuals. *Genetics in medicine : official journal of the American College of Medical Genetics*, *21*(1), 233–242. https://doi.org/10.1038/s41436-018-0013-9
