# Supplementary material for: Case Report: An Infant With Kabuki Syndrome, Alobar Holoprosencephaly and Truncus Arteriosus: A Case for Whole Exome Sequencing in Neonates With Congenital Anomalies
Source: Front Genet. 2021 Nov 25;12:766316. doi: 10.3389/fgene.2021.766316 (PMC8660850; doi:10.3389/fgene.2021.766316)
Supplement: Supplementary file 3 [file DataSheet3.PDF]

Supplementary table 2: List of genes pulled by the phenotypic filter and analyzed in the whole exome sequencing

AARS2	ABCC6	ACD	ACE	ACTA1
ACTA2	ACTB	ACTC1	ACVR2B	ACVRL1
ADAMTS3	ADCK3	ADGRG6	AEBP1	AGA
AGRN	AGT	AGTR1	AK9	AKT1
AKT3	ALDH18A1	ALDH3A2	ALG9	ALPL
ALX3	ALX4	AMER1	ANK1	ANKLE2
ANKRD11	ANTXR1	AP3B1	APC2	ARFGEF2
ARHGAP31	ARID1B	ARID2	ARSB	ARVCF
ARX	ASCC1	ASPM	ASXL1	ATP5F1A
ATP6V0A2	ATP6V1A	ATP6V1B2	ATP6V1E1	ATP7A
ATR	ATRIP	ATRX	B3GALNT2	B3GALT6
B3GAT3	B3GLCT	B4GALT7	B4GAT1	B9D1
B9D2	BAZ1B	BCOR	BCR	BLM
BMP2	BMP4	BMP7	BMPER	BMPR2
BNC2	BRAF	BRCA1	BRCA2	BRIP1
BUB1	BUB1B	BUB3	CACNA1C	CASK
CBL	CC2D2A	CCBE1	CCDC22	CCNO
CCNQ	CD96	CDC45	CDC5L	CDC6
CDH11	CDH19	CDK10	CDK5RAP2	CDK6
CDKL5	CDKN1C	CDON	CDT1	CENPE
CENPF	CENPJ	CEP120	CEP135	CEP152
CEP290	CEP55	CEP57	CEP63	CFC1
CHD1L	CHD4	CHD7	CHN2	CHRNA1
CHRNB1	CHRND	CHRNE	CHRNG	CHST14
CHST3	CKAP2L	CLCN7	CLIP2	CLPTM1
COL10A1	COL11A1	COL11A2	COL13A1	COL18A1
COL1A1	COL2A1	COL3A1	COL4A1	COL5A1

COL6A1	COL6A2	COL9A1	COL9A2	COL9A3
COLEC10	COLEC11	COMT	COQ4	COQ7
COX7B	CPLANE1	CPLX1	CPT2	CREBBP
CRELD1	CRIPT	CRKL	CSPP1	CTBP1
CTC1	CTNND2	CWC27	CYP26B1	DACT1
DAG1	DCHS1	DEAF1	DES	DHCR24
DHCR7	DHODH	DIAPH1	DIS3L2	DISP1
DKC1	DLL1	DLL3	DMP1	DNAH11
DNAH5	DNAI1	DNM1L	DOCK6	DOK7
DONSON	DPF2	DPH1	DSG2	DSP
DSTYK	DTNA	DVL1	DVL3	DYM
DYNC2H1	EBP	EFEMP2	EFNA4	EFNB1
EFTUD2	EHMT1	EIF2AK3	ELN	ENPP1
EOGT	EP300	EPG5	ERCC2	ERCC3
ERCC4	ERCC5	ERCC6	ERF	ERLIN1
ESCO2	ESR1	ETFA	ETFB	ETFDH
EVC	EVC2	EXT1	EYA1	EZH2
FAM20C	FANCA	FANCB	FANCC	FANCD2
FANCE	FANCF	FANCG	FANCI	FANCL
FANCM	FASTKD2	FAT4	FBLN5	FBN1
FBN2	FGD1	FGF10	FGF20	FGF3
FGF4	FGF8	FGFR1	FGFR2	FGFR3
FGFRL1	FH	FHL1	FIG4	FKRP
FKTN	FLI1	FLII	FLNA	FLNB
FOXC1	FOXC2	FOXE1	FOXF1	FOXH1
FOXP1	FRAS1	FREM1	FREM2	FTO
FUZ	FZD2	G6PC3	GABRD	GALNS
GAS1	GATA1	GATA3	GATA4	GATA5
GATA6	GBA	GDF1	GDF3	GDF6
GHR	GJA1	GJA5	GLB1	GLDN

GLE1	GLI2	GLI3	GLIS3	GMNN
GMPPB	GNAI3	GNE	GNPTAB	GP1BB
GPC3	GPC4	GPC6	GPKOW	GREB1L
GREM1	GRHL3	GRIP1	GTF2E2	GTF2H5
GTF2I	GTF2IRD1	GUSB	HAAO	HACD1
HCCS	HDAC8	HESX1	HGSNAT	HIC1
HINT1	HIRA	HMGB3	HNF1B	HNRNPK
HOXA1	HOXA13	HOXA4	HOXB6	HOXD13
HPSE2	HRAS	HSD17B4	HSPG2	HUWE1
HYLS1	IARS2	ICK	IDS	IDUA
IER3IP1	IFIH1	IFNG	IFT122	IFT140
IFT172	IFT43	IFT52	IFT80	IFT81
IFT88	IGBP1	IGF1R	IGF2	IL11RA
IMPAD1	INPP5E	INPPL1	INSR	INTU
INVS	IQSEC2	IRF6	IRX5	ITGA7
ITGA8	JAG1	JMJD1C	KANSL1	KAT6A
KAT6B	KATNB1	KCNAB2	KCNH1	KCNJ2
KCNK9	KCTD1	KDM5C	KDM6A	KIAA0586
KIAA1279	KIF11	KIF14	KIF7	KLF4
KLHL40	KLHL41	KLHL7	KMT2A	KMT2D
KNL1	KRAS	KYNU	L1CAM	LARGE1
LBR	LEFTY2	LEMD3	LETM1	LGI4
LHX3	LHX4	LIFR	LIG4	LIMK1
LMNA	LMOD3	LONP1	LRP2	LRP4
LRP5	LTBP2	LTBP4	MAD2L2	MAF
MAFB	MAN2B1	MAP2K1	MAP2K2	MAP3K20
MAP3K7	MAPK1	MAPRE2	MASP1	MBD5
MBTPS2	MCPH1	MCTP2	MECP2	MED12
MED13L	MED17	MEGF8	MEIS2	MFSD2A
MGAT2	MID1	MIR17HG	MKKS	MKS1

MLXIPL	MN1	MOGS	MPLKIP	MRE11
MRFACD	MSMO1	MSX1	MSX2	MUC1
MUSK	MVK	MYCN	MYH3	MYH6
MYH7	MYH9	MYL2	MYOD1	MYOT
MYPN	MYRF	NAA10	NAGLU	NALCN
NBN	NDE1	NDUFB11	NEB	NECTIN1
NEK1	NEK8	NEK9	NELFA	NF1
NFATC1	NFIA	NFIX	NHEJ1	NIN
NIPBL	NKX2-5	NKX2.5	NKX2.6	NODAL
NOG	NOTCH1	NOTCH2	NOTCH3	NPHP1
NPHP3	NPR2	NR2E1	NR2F2	NRAS
NSD1	NSD2	NSDHL	NSUN2	NUBPL
NUDT6	NUP107	NUP88	NXN	OCRL
OFD1	ORC1	ORC4	ORC6	OTUD6B
OTX2	P4HB	PACS1	PAFAH1B1	PAH
PALB2	PARN	PAX2	PAX3	PBX1
PCLO	PCNT	PCSK5	PDE4D	PDGFRB
PEX1	PEX10	PEX11B	PEX12	PEX13
PEX14	PEX16	PEX19	PEX19	PEX2
PEX26	PEX3	PEX5	PEX6	PEX7
PHC1	PHEX	PHF8	PHGDH	PIEZO2
PIGL	PIGN	PIGO	PIGT	PIGV
PIK3CA	PIK3R2	PKD1L1	PKHD1	PLCB4
PLEKHG2	PLK4	PNKP	POLR1C	POLR1D
POLR3A	POMGNT1	POMGNT2	POMK	POMT1
POMT2	POP1	POR	PORCN	POU1F1
PPP1CB	PPP1R15B	PPP1R21	PPP3CA	PQBP1
PRDM16	PRKAB2	PRKAR1A	PRKG1	PROP1
PROSIT240	PRRX1	PSAT1	PTCH1	PTCH2
PTDSS1	PTEN	PTH1R	PTPN11	PTRF

PUF60	PYCR1	QARS	RAB23	RAB3GAP1
RAB3GAP2	RAD21	RAD51	RAD51C	RAF1
RAI1	RAPSN	RARB	RARS2	RB1
RBBP8	RBM10	RBM8A	RBPJ	RECQL4
REN	RERE	RET	RFC2	RFWD3
RFX6	RIPK4	RIPPLY2	RIT1	RLIM
RMRP	RNASEH2A	RNASEH2B	RNASEH2C	RNF113A
RNU4ATAC	ROBO2	ROR2	RPGRIP1	RPGRIP1L
RPL4	RPS19	RPS26	RPS6KA3	RPSA
RREB1	RSPRY1	RTEL1	RTTN	RUNX2
RYR1	SALL1	SALL4	SAMHD1	SASS6
SATB2	SC5D	SC5DL	SCARF2	SCN1B
SCN4A	SCN5A	SEC23A	SEC24C	SEC24D
SELENON	SEMA3E	SEMA5A	SETBP1	SETD2
SETD5	SF3B4	SGCA	SH3PXD2B	SHANK3
SHH	SHOC2	SIN3A	SIX1	SIX2
SIX3	SIX5	SKI	SLC12A6	SLC17A5
SLC1A4	SLC25A1	SLC25A19	SLC25A24	SLC26A2
SLC29A3	SLC2A10	SLC39A8	SLC9A6	SLX4
SMAD2	SMAD3	SMAD4	SMAD6	SMARCAL1
SMC1A	SMC3	SMCHD1	SMO	SNAP29
SNRPB	SNX10	SNX3	SON	SOS1
SOS2	SOX10	SOX11	SOX17	SOX2
SOX5	SOX6	SOX7	SOX9	SPATA5
SPECC1L	SPEG	SPRED1	SPRTN	SPRY1
SPRY4	SRCAP	SRD5A3	SRGAP1	STAC3
STAG2	STAMBP	STAT3	STIL	STRA6
SUFU	SUMO1	TAF1	TALDO1	TAPT1
TBC1D20	TBC1D24	TBCE	TBL2	TBX1
TBX15	TBX18	TBX2	TBX20	TBX22

TBX3	TBX4	TBX5	TCAP	TCF4
TCIRG1	TCOF1	TCTN2	TCTN3	TDGF1
TERT	TFAP2A	TFAP2B	TGDS	TGFB1
TGFB2TGFB3	TGFBR1	TGFBR2	TGIF1	THEMIS
THOC6	TINF2	TLK2	TLL1	TMCO1
TMEM107	TMEM216	TMEM231	TMEM237	TMEM260
TMEM67	TMEM70	TMEM94	TNFSF11	TNNT2
TP63	TPM2	TPM3	TRAF7	TRAIP
TRAP1	TRAPPC2	TREX1	TRIP11	TRIP13
TRIP4	TRMT10A	TRPS1	TRPV4	TSC1
TSC2	TSEN2	TSEN34	TSEN54	TTC37
TTN	TUBB	TUBB2B	TUBB3	TUBGCP4
TUBGCP6	TWIST1	TWIST2	TXNL4A	UBE2T
UBE3B	UBR1	UFD1	UMOD	UPF3B
UPK3A	UQCRB	USP9X	VAC14	VANGL1
VCL	VHL	VPS13B	VPS33B	VRK1
WASHC5	WDPCP	WDR19	WDR34	WDR35
WDR60	WDR62	WDR73	WDR81	WHCR
WNT3	WNT4	WNT5A	WNT7A	WT1
XRCC2	XRCC4	XYLT1	XYLT2	YWHAE
ZBTB20	ZDHHC9	ZEB2	ZFPM2	ZIC1
ZIC2	ZIC3	ZMPSTE24	ZNF148	ZNF335
ZNF469	ZSWIM6			
